# Supplementary material for: The repurposed use of anesthesia machines to ventilate critically ill patients with Coronavirus Disease 2019 (COVID-19)
Source: Res Sq. 2021 Feb 12:rs.3.rs-228821. Preprint. [Version 1] doi: 10.21203/rs.3.rs-228821/v1 (PMC7885930; doi:10.21203/rs.3.rs-228821/v1)
Supplement: Supplement [file 6db4acd1f1179c354111cb99.docx]

**Supplemental Digital Content 1**

**ICU Care and COVID-19 Specific Therapies**

Individualized lung-protective ventilation was used, as defined by currently accepted guidelines. ^1,2^ Briefly, a tidal volume of 6 ml/kg of the predicted body weight was set, with the highest positive end-expiratory pressure (PEEP) generating a plateau pressure lower than 30 CmH_2_O and a driving pressure (dP= plateau pressure-PEEP) lower than 12 cmH_2_O^3^. The lowest possible fraction of inspired oxygen (FiO_2_) was maintained to achieve PaO_2_, or SpO_2_, targets of 55 mmHg, or 88%, respectively. Hypercapnia was permissibly tolerated, with a maximum respiratory rate of 30 breaths/min and a pH of 7.3 as limits. Pronation was considered during the first 48 hours for patients with a PaO_2_/FiO_2_ lower than 150. As per institutional practice and based on clinical judgment, lung recruitment maneuvers (LRM) were performed with one or more of the following criteria:

- body mass index higher than 30 Kg/m^2^;

- paO_2_/FiO_2_ lower than 150 with pronation unfeasible;

- persistent degree severe hypoxemia despite pronation.

LRMs were performed in pressure-controlled ventilation at driving pressure of 15 cmH_2_O, by a stepwise increase in PEEP 5 cmH_2_O every 30 seconds, targeting a maximum plateau pressure of 40 cmH_2_O held for 1 min. A decremental PEEP titration was performed in volume-controlled ventilation until the level with the lowest dP and the highest static compliance was identified. The "best PEEP" was then set with an additional +2 cmH_2_O. ^4,5^ The artificial airway was clamped in case any intervention requiring disconnection from the ventilator was deemed necessary.

On a case-by-case basis, inhaled nitric oxide was considered as a rescue strategy for refractory hypoxemia. The use of veno-venous extracorporeal membrane oxygenation (VV-ECMO) was limited to patients younger than 60 years old and only if applicable within the first ten days of mechanical ventilation.

Full coverage of the makeshift OR-ICU was provided by the same medical, nursing, and support staff of the regular ICU. Physicians working in every ICU institution-wide are highly trained individuals in the field of anesthesia and critical care who routinely work with both anesthesia machines and ICU ventilators. The personnel to patients ratio during the day were as follows:

**Standard-ICU (27 beds)**

Physician-to-patient: Morning 1:4.5 - Afternoon 1:5.5 - Night 1:9

Nurse-to-patient: Morning 1:2 - Afternoon 1:3 - Night 1:3

**OR-ICU (15 beds):**

Physicians: Morning 1:4 - Afternoon 1:5 - Night 1:7.5

Nurses: Morning 1:2 - Afternoon 1:2 - Night 1:2

No intended differences were planned with respect to physician staffing, while a slightly increased nurse-to-patient ratio was preferred in the OR-ICU due to logistical difference (5 OR ICUs of two beds each and a PACU of 5 beds making up the OR ICU compared with the normal open space in the standard ICU).

*COVID-19 Specific Therapies*

Indications for the administration of COVID-19 targeted therapies were based on the minimal evidence available during the early stages of the pandemic, uniformly applied throughout the institution^2^. Initially, lopinavir/ritonavir and hydroxychloroquine were prescribed for all patients. Later on, according to evidence becoming increasingly available, the use of lopinavir/ritonavir was suspended in favor of compassionate use remdesivir^6,7^. Additionally, according to an institutional protocol, tocilizumab was used in selected cases as an anti-interleukin-6 (IL-6) agent.^8^ Steroids, such as methylprednisolone 1-2 mg/kg for 5-7 days, were used in moderate to severe ARDS or in case of evidence of fibrotic evolution at the computed tomography scan.^2,9^

**References**

1. Fan E, Del Sorbo L, Goligher EC, Hodgson CL, Munshi L, Walkey AJ, Adhikari NKJ, Amato MBP, Branson R, Brower RG, Ferguson ND, Gajic O, Gattinoni L, Hess D, Mancebo J, Meade MO, McAuley DF, Pesenti A, Ranieri VM, Rubenfeld GD, Rubin E, Seckel M, Slutsky AS, Talmor D, Thompson BT, Wunsch H, Uleryk E, Brozek J, Brochard LJ: An Official American Thoracic Society/European Society of Intensive Care  Medicine/Society of Critical Care Medicine Clinical Practice Guideline: Mechanical  Ventilation in Adult Patients with Acute Respiratory Distress Syndrome. *Am J Respir Crit Care Med* 2017; 195:1253–63

2. Alhazzani W, Møller MH, Arabi YM, Loeb M, Gong MN, Fan E, Oczkowski S, Levy MM, Derde L, Dzierba A, Du B, Aboodi M, Wunsch H, Cecconi M, Koh Y, Chertow DS, Maitland K, Alshamsi F, Belley-Cote E, Greco M, Laundy M, Morgan JS, Kesecioglu J, McGeer A, Mermel L, Mammen MJ, Alexander PE, Arrington A, Centofanti JE, Citerio G, [Bandar Baw](https://pubmed.ncbi.nlm.nih.gov/?term=Baw+B&cauthor_id=32222812) , [Ziad A Memish](https://pubmed.ncbi.nlm.nih.gov/?term=Memish+ZA&cauthor_id=32222812), [Naomi Hammond](https://pubmed.ncbi.nlm.nih.gov/?term=Hammond+N&cauthor_id=32222812), [Frederick G Hayden](https://pubmed.ncbi.nlm.nih.gov/?term=Hayden+FG&cauthor_id=32222812), [Laura Evans](https://pubmed.ncbi.nlm.nih.gov/?term=Evans+L&cauthor_id=32222812), [Andrew Rhodes](https://pubmed.ncbi.nlm.nih.gov/?term=Rhodes+A&cauthor_id=32222812): Surviving Sepsis Campaign: guidelines on the management of critically ill adults  with Coronavirus Disease 2019 (COVID-19). Intensive Care Med 2020; 46:854–87

3. Amato MBP, Meade MO, Slutsky AS, Brochard L, Costa ELV, Schoenfeld DA, Stewart TE, Briel M, Talmor D, Mercat A, Richard J-CM, Carvalho CRR, Brower RG: Driving Pressure and Survival in the Acute Respiratory Distress Syndrome. *N Engl J Med 2015*; 372:747–55

4. Barbas CSV, Matos GFJ de, Okamoto V, Borges JB, Amato MBP, Carvalho CRR de: Lung recruitment maneuvers in acute respiratory distress syndrome. *Respir Care Clin N Am* 2003; 9:401–18, vii

5. Keenan JC, Formenti P, Marini JJ: Lung recruitment in acute respiratory distress syndrome:  what is the best strategy? *Current Opinion in Critical Care* 2014; 20

6. B. Cao, Y. Wang, D. Wen, W. Liu, Jingli Wang, G. Fan, L. Ruan, B. Song, Y. Cai, M. Wei, X. Li, J. Xia, N. Chen, J. Xiang, T. Yu, T. Bai, X. Xie, L. Zhang, C. Li, Y. Yuan, H. Chen, Huadong Li, H. Huang, S. Tu, F. Gong, Y. Liu, Y. Wei, C. Dong, F. Zhou, X. Gu, J. Xu, Z. Liu, Y. Zhang, Hui Li, L. Shang, K. Wang, K. Li, X. Zhou, X. Dong, Z. Qu, S. Lu, X. Hu, S. Ruan, S. Luo, J. Wu, L. Peng, F. Cheng, L. Pan, J. Zou, C. Jia, Juan Wang, X. Liu, S. Wang, X. Wu, Q. Ge, J. He, H. Zhan, F. Qiu, L. Guo, C. Huang, T. Jaki, F.G. Hayden, P.W. Horby, D. Zhang, and C. Wang: A Trial of Lopinavir–Ritonavir in Adults Hospitalized with Severe Covid-19. N Engl J Med 2020; 382:1787–99

7. J. Grein, N. Ohmagari, D. Shin, G. Diaz, E. Asperges, A. Castagna, T. Feldt, G. Green, M.L. Green, F.-X. Lescure, E. Nicastri, R. Oda, K. Yo, E. Quiros-Roldan, A. Studemeister, J. Redinski, S. Ahmed, J. Bernett, D. Chelliah, D. Chen, S. Chihara, S.H. Cohen, J. Cunningham, A. D’Arminio Monforte, S. Ismail, H. Kato, G. Lapadula, E. L’Her, T. Maeno, S. Majumder, M. Massari, M. Mora-Rillo, Y. Mutoh, D. Nguyen, E. Verweij, A. Zoufaly, A.O. Osinusi, A. DeZure, Y. Zhao, L. Zhong, A. Chokkalingam, E. Elboudwarej, L. Telep, L. Timbs, I. Henne, S. Sellers, H. Cao, S.K. Tan, L. Winterbourne, P. Desai, R. Mera, A. Gaggar, R.P. Myers, D.M. Brainard, R. Childs, and T. Flanigan: Compassionate Use of Remdesivir for Patients with Severe Covid-19. N Engl J Med 2020; 382:2327–36

8. Xu X, Han M, Li T, Sun W, Wang D, Fu B, Zhou Y, Zheng X, Yang Y, Li X, Zhang X, Pan A, Wei H: Effective treatment of severe COVID-19 patients with tocilizumab. *Proc Natl Acad Sci* USA 2020; 117:10970

9. Wu Z, McGoogan JM: Characteristics of and Important Lessons From the Coronavirus Disease 2019 (COVID-19) Outbreak in China: Summary of a Report of 72 314 Cases From the Chinese Center for Disease Control and Prevention. *JAMA* 2020; 323:1239–42
