# Supplementary material for: The repurposed use of anesthesia machines to ventilate critically ill patients with Coronavirus Disease 2019 (COVID-19)
Source: Res Sq. 2021 Feb 12:rs.3.rs-228821. Preprint. [Version 1] doi: 10.21203/rs.3.rs-228821/v1 (PMC7885930; doi:10.21203/rs.3.rs-228821/v1)
Supplement: Supplement [file e10f24c85beb3d24465d8ea1.docx]

**Supplemental Digital Content 2**

**Table 1: Cause of Death**

|  |  | | |  |  |  |  |  |  |  |  |
| --- | --- | --- | --- | --- | --- | --- | --- | --- | --- | --- | --- |
| **Patient** | | **AM** | **Age,** *years* | | **Sex** | **BMI** | **APACHE II** | **Barotrauma during mechanical ventilation^¶^** | **Acute Airway Obstruction during mechanical ventilation^§^** | **ICU Length of Stay,** *days* | **Main Cause of Death** |
| 1 | | Yes | 65 | | M | 32 | 14 | No | No | 6 | Refractory Septic Shock |
| 2 | | Yes | 71 | | M | 29 | 16 | No | No | 4 | Refractory Hypoxia |
| 3 | | Yes | 62 | | F | 28 | 9 | No | No | 45 | Refractory Hypoxia |
| 4 | | Yes | 65 | | F | 26 | 19 | No | No | 7 | Acute Pulmonary Thromboembolism |
| 5 | | Yes | 71 | | M | 28 | 14 | No | No | 3 | Refractory Hypoxia |
| 6 | | Yes | 50 | | M | 25 | 17 | No | Yes | 53 | Refractory Hypoxia |
| 7 | | Yes | 70 | | M | 28 | 17 | Yes | No | 17 | Refractory Hypoxia |
| 8 | | Yes | 64 | | M | 26 | 18 | No | No | 8 | ARDS / Bacterial Infection |
| 9 | | Yes | 40 | | M | 24 | 4 | No | Yes | 28 | ARDS / Bacterial Infection |
| 10 | | Yes | 73 | | F | 35 | 15 | No | No | 12 | Ischemic Bowel Disease |
| 11 | | Yes | 70 | | M | 24 | 14 | Yes | No | 6 | ARDS / Septic Shock |
| 12 | | Yes | 59 | | M | 26 | 12 | No | Yes | 14 | Acute Airway Obstruction |
| 13 | | No | 49 | | M | 26 | 8 | No | No | 9 | Refractory Septic Shock |
| 14 | | No | 68 | | M | 22 | 18 | No | No | 18 | Refractory Hypoxia |
| 15 | | No | 59 | | M | 25 | 7 | No | No | 18 | ARDS / Right Ventricular Failure |
| 16 | | No | 67 | | M | 27 | 14 | No | No | 12 | Refractory Hypoxia |
| 17 | | No | 68 | | M | 31 | 10 | No | No | 10 | ARDS / Bacterial Infection |
| 18 | | No | 62 | | F | 26 | 12 | No | No | 6 | Refractory Hypoxia |
| 19 | | No | 75 | | M | 44 | 26 | No | No | 7 | Refractory Hypoxia |
| 20 | | No | 59 | | M | 40 | 11 | No | No | 20 | Refractory Septic Shock* |
| 21 | | No | 64 | | M | 26 | 13 | No | No | 33 | Refractory Hypoxia |
| 22 | | No | 72 | | M | 31 | 15 | No | No | 3 | Refractory Hypoxia |
| 23 | | No | 63 | | M | 28 | 13 | No | No | 13 | Refractory Septic Shock |
| 24 | | No | 68 | | M | 31 | 17 | No | No | 13 | ARDS / Bacterial Infection |
| 25 | | No | 64 | | M | 26 | 13 | No | No | 20 | Refractory Septic Shock |
| 26 | | No | 54 | | M | 31 | 12 | No | No | 58 | Refractory Hypoxia |
| 27 | | No | 55 | | M | 25 | 28 | No | No | 12 | Refractory Septic Shock |
| 28 | | No | 61 | | M | 40 | 11 | No | No | 9 | Refractory Hypoxia |
| 29 | | No | 67 | | M | 35 | 11 | No | No | 6 | Refractory Septic Shock |
| 30 | | No | 27 | | M | 33 | 5 | No | No | 19 | Refractory Hypoxia |
| 31 | | No | 73 | | M | 28 | 11 | No | No | 20 | Refractory Hypoxia / Myocardial Infarction |
| 32 | | No | 64 | | F | 35 | 9 | Yes | No | 24 | Refractory Hypoxia |
| 33 | | No | 62 | | M | 28 | 20 | No | No | 5 | Multiple Organ Failure |
| 34 | | No | 70 | | M | 31 | 14 | No | No | 5 | Refractory Hypoxia |
| 35 | | No | 58 | | M | 32 | 5 | No | No | 4 | Refractory Hypoxia |
| 36 | | No | 70 | | M | 43 | 16 | No | No | 5 | Refractory Hypoxia |
| 37 | | No | 65 | | M | 24 | 13 | No | No | 11 | Refractory Hypoxia |
| 38 | | No | 53 | | M | 28 | 6 | No | No | 15 | Refractory Hypoxia |
| 39 | | No | 33 | | M | 36 | 8 | No | No | 14 | Refractory Septic Shock |

***Abbreviations****: AM = Allocation to Anesthesia Machine; BMI = Body Mass Index; APACHE II = Acute Physiology and Chronic Health Disease Classification System II; ICU = Intensive Care Unit; ARDS = acute respiratory distress syndrome.*

*¶ Barotrauma has been defined as* *spontaneous pneumothorax and/or pneumomediastinum during invasive mechanical ventilation.*

*§ Extubation and immediate re-intubation due to life-threatening airway occlusion*

**Patient Died After ICU discharge*

**Table 2: Univariate Cox Regression Analysis for 60-day Mortality**

| Variable | Hazard ratio | 95% Confidence Interval | P value |
| --- | --- | --- | --- |
| Female sex | 0.46 | 0.18-1.17 | 0.104 |
| Anesthesia machine^§^ | 2.46 | 1.25-4.87 | 0.010 |
| Age at admission, per year^§^ | 1.06 | 1.03-1.10 | 0.001 |
| BMI, per kg/m^2^ | 1.04 | 0.98-1.10 | 0.198 |
| Hypertension^§^ | 2.34 | 1.20-4.56 | 0.013 |
| Diabetes mellitus^§^ | 2.29 | 1.11-4.72 | 0.024 |
| Obesity | 1.59 | 0.83-3.06 | 0.165 |
| COPD^§^ | 5.85 | 2.23-15.30 | < 0.001 |
| Hypercholesterolemia^§^ | 2.08 | 0.98-4.38 | 0.055 |
| Bilirubin, per mg/dl^§^ | 1.48 | 1.18-1.86 | 0.001 |
| Creatinine, per mg/dl^§^ | 4.67 | 2.33-9.35 | < 0.001 |
| Lactate, per mmol/L^§^ | 1.96 | 1.35-2.84 | < 0.001 |
| D-dimer, per mg/dl | 1.02 | 0.99-1.05 | 0.115 |
| Mean arterial pressure, per mmHg^§^ | 0.96 | 0.93-0.99 | 0.008 |
| PEEP, per cmH_2_O | 1.12 | 0.97-1.31 | 0.133 |
| P_plat,_ per cmH_2_O | 0.94 | 0.85-1.05 | 0.272 |
| Driving Pressure, per cmH_2_O^§^ | 0.89 | 0.78-1.01 | 0.071 |
| Tidal Volume/PBW, per ml/kg | 0.81 | 0.53-1.22 | 0.308 |
| RR, per breath | 1.02 | 0.94-1.12 | 0.596 |
| PaO_2_, per mmHg | 1.00 | 0.99-1.01 | 0.729 |
| PaCO_2_, per mmHg | 1.02 | 0.99-1.04 | 0.165 |
| pH, per unit^§^ | 0.02 | 0.00-0.44 | 0.015 |
| Heart rate, per beat | 0.99 | 0.98-1.01 | 0.521 |
| WBC, per 10^9^/L | 0.99 | 0.93-1.05 | 0.674 |
| Hematocrit, per %^§^ | 1.11 | 1.04-1.19 | 0.003 |
| Hemoglobin, per mg/dl^§^ | 1.37 | 1.11-1.68 | 0.003 |
| Platelets, per 10^12^/L | 1.00 | 0.99-1.00 | 0.155 |
| Corticosteroids | 0.59 | 0.30-1.17 | 0.132 |
| Tocilizumab | 0.69 | 0.34-1.38 | 0.294 |
| Remdesivir | 0.76 | 0.27-2.14 | 0.603 |
| Prone positioning^§^ | 2.32 | 1.19-4.52 | 0.013 |
| Inhaled Nitric Oxide^§^ | 2.34 | 1.03-5.33 | 0.043 |
| PaO_2_/FiO_2_ ratio, per unit | 1.00 | 0.99-1.00 | 0.192 |

PEEP: Positive End-Expiratory Pressure; P_plat_: Plateau Pressure; PBW: Predicted Body weight; ^§^Variable considered for multivariable analysis.

**Table 3: Rates of Missing Data For Each Variable**

| Variable | N Missing (%) |
| --- | --- |
| Anesthesia machine | 0 (0) |
| Age at admission | 0 (0) |
| Sex | 0 (0) |
| Body mass index | 3 (3.4) |
| Race | 0 (0) |
| APACHE II Score | 4 (4.5) |
| SOFA Score | 28 (31.5) |
| Hypertension | 0 (0) |
| Diabetes mellitus | 1 (1.1) |
| Obesity | 1 (1.1) |
| COPD | 0 (0) |
| Hypercholesterolemia | 0 (0) |
| **Clinical variables** |  |
| PaO_2_/FiO_2_, mmHg | 5 (5.6) |
| PEEP, cmH_2_O | 3 (3.4) |
| P_plat_, cmH_2_O | 16 (18.0) |
| Vt/PBW (ml/kg) | 5 (5.6) |
| RR, breaths/min | 5 (5.6) |
| C_rs_, ml/cmH_2_O | 17 (19.1) |
| dP, cmH_2_O | 16 (18.0) |
| PaO_2_, mmHg | 5 (5.6) |
| PaCO_2_, mmHg | 5 (5.6) |
| pH | 8 (9.0) |
| HCO_3_^-^, mmol/l | 18 (20.2) |
| Base excess | 11 (12.4) |
| Lactate, mmol/l | 11 (12.4) |
| Heart rate, beats/min | 7 (7.9) |
| MAP, mmHg | 6 (6.7) |
| **Laboratory findings** |  |
| CRP, mg/dl | 14 (15.7) |
| Procalcitonin, ng/ml | 23 (25.8) |
| WBC, 10^9^/L | 5 (5.6) |
| Tot. lymphocytes, % | 12 (13.5) |
| Hematocrit, % | 6 (6.7) |
| Hemoglobin, mg/dl | 5 (5.6) |
| Platelets, 10^12^/L | 5 (5.6) |
| ALT, IU/L | 12 (13.5) |
| AST, IU/L | 17 (19.1) |
| LDH, IU/L | 18 (20.2) |
| Bilirubin, mg/dl | 17 (19.1) |
| Creatinine, mg/dl | 12 (13.5) |
| Glucose, mg/dl | 24 (27.0) |
| Sodium, mEq/l | 20 (22.5) |
| Potassium, mEq/l | 20 (22.5) |
| Creatine kinase, IU/l | 24 (27.0) |
| CK-MB, ng/ml | 42 (47.2) |
| Troponin-T, ng/l | 36 (40.4) |
| NT-proBNP, ng/l | 42 (47.2) |
| PT, s | 23 (25.8) |
| aPTT, s | 23 (25.8) |
| D-dimer, mcg/ml | 44 (39%) |
| **Treatments received, no. (%)** |  |
| Lopinavir/ritonavir | 0 (0) |
| Hydroxychloroquine | 0 (0) |
| Antibiotic prophylaxis | 0 (0) |
| Corticosteroids | 1 (1.1) |
| Tocilizumab | 1 (1.1) |
| Remdesivir | 1 (1.1) |
| Continuous IV sedation | 0 (0) |
| Continuous IV opioids | 0 (0) |
| Inhaled sedation | 0 (0) |
| Paralysis | 0 (0) |
| Prone positioning | 1 (1.1) |
| Inhaled Nitric Oxide | 1 (1.1) |
| **Outcomes** |  |
| Mortality | 0 (0) |
| ICU length of stay | 0 (0) |
| ICU-free days | 0 (0) |
| Hospital length of stay | 0 (0) |
| Hospital-free days | 0 (0) |
| Mechanical ventilation days | 0 (0) |
| Ventilator-free days | 0 (0) |
| ECMO initiation | 0 (0) |
| Tracheostomy | 0 (0) |
| Barotrauma | 0 (0) |
| Emergency tube exchange | 0 (0) |

*Abbreviations: APACHE II: Acute Physiology and Chronic Health Evaluation II; SOFA: Sequential Organ Failure Assessment; COPD: Chronic Obstructive Pulmonary Disease; PEEP: Positive End-Expiratory Pressure; P_plat_: Plateau Pressure; Vt: Tidal Volume; PBW: Predicted Body Weight; RR: Respiratory Rate; C_rs_: Respiratory System Compliance; dP: Driving Pressure (P_plat_ – PEEP); MAP: Mean Arterial Pressure; CRP: C-Reactive Protein; WBC: White Blood Cells; ALT: Alanine Aminotransferase; AST: Aspartate Aminotransferase; LDH: Lactate Dehydrogenase; NT-proBNP: N-Terminal pro B-type Natriuretic Peptide; PT: Prothrombin time; aPTT (activated partial thromboplastin time); IL-6: Interleukin-6; IV: Intravenous; ECMO: Extra-Corporeal Membrane Oxygenation; ICU: Intensive Care Unit.*

**Table 4: Frequency of Variable Inclusion in 100 Bootstrapped Samples**

| Variable | N Models Variable Included In |
| --- | --- |
| Anesthesia Machine*^§^* | 85 (85%) |
| Creatinine*^§^* | 89 (89%) |
| Hypertension*^§^* | 71 (71%) |
| Bilirubin*^§^* | 68 (68%) |
| Inhaled Nitric oxide | 63 (63%) |
| Hypercolesterolemia | 55 (55%) |
| Age | 55 (55%) |
| Diabetes mellitus | 51 (51%) |
| COPD | 50 (50%) |
| pH | 49 (49%) |
| Driving Pressure | 46 (46%) |
| Mean arterial blood pressure | 45 (45%) |
| Hematocrit | 44 (44%) |
| Hemoglobin | 44 (44%) |
| Lactate | 41 (41%) |
| *^§^Variable selected for final multivariable sensitivity analysis; Abbreviations: COPD = chronic obstructive pulmonary disorder* | |

**Table 5: Final Sensitivity Model Using Bootstrapped Samples**

| Variable | Hazard Ratio | 95% Confidence Interval | P value |
| --- | --- | --- | --- |
| Allocation to an Anesthesia Machine | 3.46 | 1.57-7.63 | 0.002 |
| Creatinine, per mg/dl | 8.37 | 3.13-22.39 | <0.001 |
| Hypertension | 3.35 | 1.46-7.69 | 0.005 |
| Bilirubin, per mg/dl | 1.52 | 1.20-1.94 | 0.001 |

**Day 1 Day 2 Day 7**

**Figure 1: Change in mean arterial pressure in the two groups (AM and ICU-VENT)**

*Data are presented at baseline, 48 hours and on day 7. Bar extremities represent the interquartile range. * p=0.05*
